# Joint congestion and contention avoidance in a scalable QoS-aware opportunistic routing in wireless ad-hoc networks

**DOI:** 10.1371/journal.pone.0288955

**Published:** 2023-08-01

**Authors:** Ali Parsa, Neda Moghim, Sasan Haghani

**Affiliations:** 1 Department of Computer Engineering, University of Isfahan, Isfahan, Iran; 2 Virginia Modeling, Analysis, and Simulation Center, Old Dominion University, Suffolk, VA, United States of America; 3 Department of Electrical and Computer Engineering, University of the District of Columbia, Washington, DC, United States of America; Khon Kaen University, THAILAND

## Abstract

Opportunistic routing (OR) can greatly increase transmission reliability and network throughput in wireless ad-hoc networks by taking advantage of the broadcast nature of the wireless medium. However, network congestion is a barrier in the way of OR’s performance improvement, and network congestion control is a challenge in OR algorithms, because only the pure physical channel conditions of the links are considered in forwarding decisions. This paper proposes a new method to control network congestion in OR, considering three types of parameters, namely, the backlogged traffic, the traffic flows’ Quality of Service (QoS) level, and the channel occupancy rate. Simulation results show that the proposed algorithm outperforms the state-of-the-art algorithms in the context of OR congestion control in terms of average throughput, end-to-end delay, and Packet Delivery Ratio (PDR). Due to the higher PDR at different traffic loads and different node densities, it can be concluded that the proposed algorithm also improves network scalability, which is very desirable given the recent changes in wireless networks.

## Introduction

Routing in wireless ad-hoc networks is a research topic that has received significant attention [1, 2]. Conventionally, routing protocols consist of two major phases: routing and forwarding. In the routing phase, network status information is exchanged between network nodes. Then, each node forms its routing table to identify its best next hops to all potential destinations in the network. The forwarding phase occurs when packets arrive, and the packets will be forwarded through the paths, specified by the routing tables. Although this approach has been widely used in ad-hoc networks, it does not make efficient use of wireless medium features such as broadcast nature and capability of receiving the packets by long-distance links [3].

Opportunistic Routing (OR) [4] has been proposed to exploit the broadcast nature of the wireless medium, which causes packets to be received not only by the intended next-hop but also by the nodes in the sender’s vicinity. In the OR paradigm, network nodes have no fixed routing table for packet forwarding. When a packet is sent on the wireless channel, it is received by the nodes in the sender’s transmission range. Usually, a subset of the recipient neighboring nodes (the Candidate Set (CS)) is selected and prioritized by the sender to participate in the forwarding process [5]. Among the CS members, the node with the highest priority forwards the packet and the others discard it. This procedure is repeated until the packet reaches its destination.

**Fig 1:**
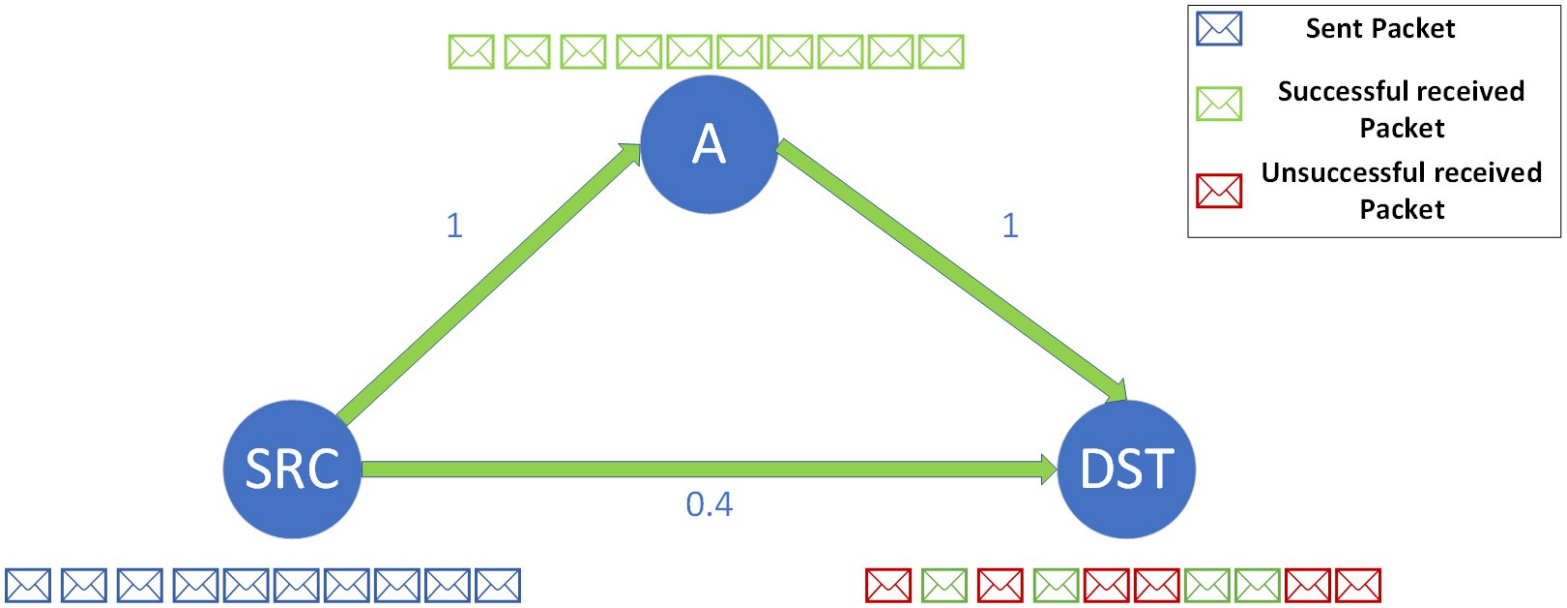
An example of the benefits of OR compared to traditional routing.

To show the benefit of the OR compared to the traditional routing, an example is presented in Fig 1. There are 3 nodes in this figure. The links between the nodes are shown with their Packet Delivery Ratio (PDR) as the numbers on them. SRC wants to send 10 data packets to DST. Based on the showed PDR values, a traditional routing algorithm selects the path of SRC-A-DST, and uses Node A as an intermediate node. However, due to the broadcast nature of the wireless transmission, some of the data packets (40% on average), which are intended to be received at Node A, are also received at the destination simultaneously. The OR takes advantage of this issue and improves the network performance.

The performance of OR has been fully investigated by many researchers [4, 6, 7]. Different OR protocols have also been proposed in diverse wireless networks, such as Wireless Sensor Networks (WSNs) [8–10], Mobile Ad-hoc Networks (MANETs) [11], Vehicular Ad-hoc Networks (VANETs) [12], and Flying Ad-hoc Networks (FANETs) [13]. Various challenges have also been raised in OR, such as CS selection, CS prioritizing rule (routing metric), discarding policy (inter-node coordination), packet duplication, mobility, and Quality of Service (QoS). Recently, some Machine Learning (ML) based methods have been proposed to solve the OR challenges [14, 15].

In this paper, we focus on QoS-related issues, especially the congestion control aspects. Since routing metrics often reflect the physical channel conditions, nodes with better link qualities gain higher forwarding priority and forward the major part of the traffic. This causes congestion on high-quality links, which leads to unbounded delays [16]. To solve this problem, some researchers have focused on traffic distribution in OR through backpressure routing policies. In other words, queue length information is considered as a sign of congestion and used in the computation of the routing metric. Therefore, when the queue length of a node becomes longer, its forwarding priority decreases. By using this policy, the nodes with lower link quality can also participate in the forwarding process. So, backpressure routing increases the number of the network’s active nodes implicitly.

Although backpressure routing mitigates the congestion problem due to the use of further nodes, it increases channel occupancy level and subsequently, access delay. In other words, this network policy tends to involve more nodes in the forwarding process for the sake of traffic distribution. Therefore, the performance of Medium Access Control (MAC) algorithms degrades due to increasing delays, such as back-off time. This problem becomes more severe with the advent of the Internet of Things (IoT) [17]. As the number of nodes increases, network access control becomes more challenging due to the contention of the network nodes. Multiple solutions have been proposed to deal with such a situation, in which the MAC mechanism is usually changed [9]. Although changing the MAC mechanism is a straightforward solution, we believe that a cross-layer approach will be more effective in the context of OR. This issue leads us to consider the channel-level condition for better traffic distribution and also network scalability improvement.

This paper aims to propose a new OR algorithm in a fixed wireless network that establishes a trade-off between traffic distribution and MAC level conditions in a multi-flow environment. Briefly, in the proposed algorithm, each node computes the overall delay of delivering packets to all other network nodes. The overall delay consists of two major components, channel access delay, and queuing delay. When a network node has a packet to send, it decides to include its neighbor in the CS if it results in a lower overall delay. The main contributions of this paper can be summarized as follow:

- Establishing a trade-off between queuing delay and channel access delay in an OR scheme
- Finding the optimal forwarding nodes in OR that improve the overall network performance
- Providing an overview of the network status for each traffic flow to select the optimal CS
- Consideration of some cross-layer issues, such as dynamic retry threshold and MAC queue awareness, to help improve the forwarding process

The rest of this paper is organized as follows. The Related Works Section provides an overview of OR, summarizes the challenges of the OR and the related research works. The main contributions of this paper are given in the Proposed Method Section. The Performance Evaluation Section presents the simulation results of the proposed algorithm and compares them to the state-of-the-art algorithms. Finally, the paper is summarized and concluded in the Conclusion Section.

## Related works

In this section, we provide an overview of the basic research works that have introduced and expanded OR. Then, some previous works that address QoS-related issues such as congestion control, bandwidth requirements, and delay requirements are briefly described. A comparison between the proposed method and the related state-of-the-art algorithms is presented and the method used for delay analysis in a multi-flow environment is also briefly introduced.

### Basic OR algorithms

The broadcast nature of the wireless medium has been exploited in earlier studies [18] by utilizing cooperative diversity, which virtually builds an array antenna through repeating the signal by multiple terminals. Extremely Opportunistic Routing (ExOR) [4] utilizes the wireless medium’s broadcast nature in the network layer. The tasks of routing and MAC have been tied together in the ExOR algorithm. After ExOR introduced the main idea of OR, it was suggested that the separation of MAC and routing can increase the efficiency of the OR. Thus, Mac-independent Opportunistic Routing and Encoding (MORE) [6] was proposed, in which routing and MAC processes were separated. Random intra-session network coding [19] is also used in MORE to solve the inter-node coordination problem. Many of the subsequent research such as [3, 5, 20, 21] have extended the basic ideas of ExOR and MORE and specialized these protocols for better performance in different situations. [22, 23] provide more details on the basic idea and also the design challenges of OR.

### Congestion control in OR

QoS-related concepts cause new challenges for OR algorithms. Congestion control is one of these important QoS-related issues that can affect the routing policy. Opportunistic Routing with Congestion Diversity (ORCD) [16] is one of the QoS-related primitive works that aim at congestion control in OR. It tries to forward data packets by the network nodes with minimum overall congestion. ORCD’s routing metric is a combination of the nodes’ queue length and Packet PDR to reflect congestion. An extended version of ORCD [24] was also proposed as Distributed Opportunistic Routing with Congestion Diversity (D-ORCD) that contains a candidate set selection method as well. Moreover, it includes more realistic computations for the routing metric. Like [16, 24], congestion control is one of the main goals of this paper. But, we use a novel approach to overcome congestion that considers channel contention level status in addition to the nodes’ queue length as a congestion indicator.

### Bandwidth requirements in OR

Meeting the bandwidth requirements of the traffic flows is another QoS-related research topic that has been investigated in OR schemes. Limiting the CS to the nodes with sufficient resources is the common solution to meet the QoS requirements of the traffic flows in terms of bandwidth. However, the path diversity of the OR should not be fully forfeited by limiting the CS. Traffic flows’ bandwidth provisioning is the main contribution of Bandwidth-aware Opportunistic Routing with Admission Control (BOR/AC) [25]. A routing metric, named Bandwidth-Cost Ratio (BCR) is proposed that consists of the available bandwidth of the nodes and the transmission cost. When a new traffic flow arrives, the call admission control process will accept the traffic flow if the network is capable of providing its bandwidth requirement. An enhanced version of BOR/AC was also proposed as the Bandwidth-aware Opportunistic Routing Algorithm (BORA) [26]. QoS provisioning is the main goal of Opportunistic Routing with Admission Control (ORAC) [27]. In ORAC, the network nodes’ capacity is computed based on their bandwidth, queue length, and residual energy. Then, call admission control accepts a traffic flow if the network can meet its requirements. Calado et al. [28] propose a call admission control to provide the bandwidth requirement of the traffic flow. After the traffic flow admission, different amounts of bandwidth will be reserved on different links based on the amount of the nodes’ participation in the forwarding process. QoS improvement in terms of throughput is another goal of this paper, like [25–28]. But this paper has a fundamental difference. Other works try to provide the best network resources for the traffic flows without considering the traffic flows’ required QoS level. However, in our method, network resources will be provided for the traffic flows based on their QoS level, not more.

### Delay requirements in OR

The delay requirement of the traffic flows is another QoS parameter that must be considered in OR. Furthermore, due to health, environmental, and economic issues, the transmission power of wireless stations cannot be greatly increased. These constraints force the stations to use as low transmission power as possible [29]. So, the quality of the wireless link will be decreased. The OR scheme will be beneficial for such situations (refer to the example shown in Fig 1) that can help to meet the users’ delay requirements. Efficient QoS-aware Geographic Opportunistic Routing (EQGOR) [30] is a research that aims at QoS provisioning in wireless ad-hoc networks. It cares about the end-to-end delay and reliability requirements of the traffic flows. CS limitation is the main tool to achieve these goals, which is performed in a hop-by-hop manner. In each hop, the remaining part of the required delay is calculated based on the elapsed time duration from the packet’s transmission; and accordingly, the remaining hop count to the ultimate destination is estimated. Then, a multi-objective multi-constraint optimization problem is formed according to the obtained information with the main objective of maximizing the SPEED [31] parameter. Like EQGOR, providing end-to-end delay and reliability are the main objectives of the QoS-aware Multi-sink Opportunistic Routing (QMOR) [32]. A multi-objective multi-constraint optimization problem is proposed to formulate the problem. The main objective of the optimization problem is to minimize the transmission cost, with the constraints of the traffic flows’ QoS requirements. This paper has formulated the candidate set selection as an optimization problem like [30, 32]. However, the optimization problem selects the best candidate set for each packet in a hop-by-hop manner and based on its belonged traffic flow.

### Performance analysis of 802.11 DCF

In multi-flow environments, a network node probably participates in the forwarding process of multiple traffic flows. This makes node-to-node analysis of the network performance very difficult. Thus, implicit methods are used to model the network conditions. Mathematical models can provide insight into the network condition and therefore, can be used for QoS-related operations. Bianchi’s model [33] is one of the early works in the area of the performance evaluation of the IEEE 802.11 standard. Other researchers have extended Bianchi’s model and have changed it to adapt to various conditions, such as various traffic patterns, different numbers of nodes, and various network topologies, e.g., [34–40]. As mentioned before, MAC-related delays, such as backoff time form a major part of the end-to-end delay. In a multi-traffic flow environment, multiple traffic flows may pass through a specific node in the network. Therefore, the information of all traffic flows and their behavior should be considered in the delay computation to provide an overall point of view of the network. We use 802.11 DCF analysis, instead of per-flow analysis in the network access delay computation for more simplicity.

Table 1 provides a comparison of some previous research, most similar to the proposed method in this paper, in terms of congestion control, QoS enhancement, and candidate set optimization problem.

**Table 1:** Related works comparison.

Similarity Factor	Congestion Control	QoS Enhancement		Candidate Set Optimization		
Algorithms	DORCD [24]	BORA [26]	ORAC [27]	EQGOR [30]	QMOR [32]	Proposed Algorithm
Controlling the congestion	✓	×	✓	×	×	✓
Considering delay requirements	×	×	×	✓	✓	✓
Considering bandwidth requirements	×	✓	✓	×	×	✓
Considering the nodes’ queue length	✓	×	✓	×	×	✓
Considering channel contention level status	×	✓	×	×	×	✓

## Proposed method

As mentioned in the Introduction Section, QoS-aware congestion control is the main goal of this paper, where we propose a novel OR algorithm that distributes the traffic across the network considering link quality, network occupancy level, nodes’ queue length, and the traffic flows’ QoS level. It is worth noting that the proposed algorithm will be more efficient when the network is in the saturation state, where its traffic load is heavy.

Routing policies are the main tool for network-assisted congestion control which is interpreted as the CS selection and prioritization in the context of OR. To perform a QoS-aware CS selection and prioritization, two types of information are needed; first, QoS levels of the traffic flows, and second, the network nodes’ forwarding quality. The former can be obtained using a labeling mechanism, in which the source node attaches labels to its packets. These labels show the traffic flows’ QoS level and also their priority. For the latter, a discriminating parameter, called routing metric is needed that reflects the forwarding quality of the nodes. The routing metric of the proposed algorithm should cover both intrinsic network-related parameters, such as link quality, as well as workload parameters, such as nodes’ traffic load and network occupancy level.

For the sake of consistency and to simplify the framework, the delay is selected to show both the traffic flows’ QoS level and the forwarding quality of the nodes. This means that the source node tags the packets with the maximum tolerable delay of their corresponding traffic flows, which represents their QoS level. Delays between each node and the other network nodes, as potential destinations, are computed and used to show the nodes’ forwarding quality. Network occupancy level and nodes’ queue length also take part in the delay computation. The main components of the proposed algorithm can be summarized as follows:

- A labeling mechanism is proposed to indicate the QoS level of the traffic flows.
- The routing metric is calculated to indicate the forwarding quality of the network nodes.
- The CS members are selected and prioritized based on the computed routing metric.
- Some cross-layer procedures such as MAC-queue aware packet retransmission and dynamic retry threshold are utilized to improve the performance of the proposed algorithm.

The remainder of this section will explain the details of the proposed algorithm including the four above-mentioned procedures, and then the operation of the network node when facing different events will be explained. Therefore, the first and the second subsections describe the operation of the proposed algorithm. The third subsection denotes the routing metric calculations and the fourth subsection explains the CS selection method. Finally, the fifth subsection explains some cross-layer operations to improve the performance of the proposed algorithm.

### Forwarding scheme as the network layer behavior

The forwarding scheme describes how source, potential forwarder, and destination nodes operate. The forwarding scheme should be clearly explained to make the delay estimation more intelligible. In this section, it is assumed that the routing metric has been already calculated, and therefore, each network node has a matrix indicating the estimated delay between each possible node pair in the network. An important assumption in the proposed algorithm is that the wireless ad-hoc network is assumed to be stationary and the nodes do not have any movement. The forwarding scheme used in our proposed algorithm will be explained in three parts that show the way the source node, intermediate nodes, and destination node behave. The explained forwarding scheme is derived from [30].

- **Source node**: The source node selects a list of the candidate nodes and sorts them based on their routing metrics to the destination. Then, the source appends the ordered list as CS to the packet’s header and sends it. The source then awaits to receive acknowledgment (ACK) messages from the CS members. When the first ACK is received, it can send its next packet. Otherwise, if no ACK is received at a given period, the source node retransmits the packet. The maximum number of packet retransmissions is limited to a specific threshold called the maximum retransmission limit and then, the packet is discarded.
- **Intermediate nodes**: Each node that receives the sent packet checks the CS in the packets’ header. If the node’s ID exists in the candidate list of the packet, it will send an ACK to the sender after a specified waiting period. The waiting period is set based on the node’s priority in the CS. Since all packets are sent in the broadcast mode, lower priority forwarders likely hear the ACK sent by the higher priority ones. If a candidate forwarder receives an ACK for its last received packet during its waiting period, it will infer that a higher priority forwarder has accepted the forwarding of the packet. So, it will refrain from sending ACK and will discard the received packet. Otherwise, if no ACK is received, the node will forward the packet in a way similar to the sender, and at the end of its waiting time. This process will continue until the packet reaches its destination.
- **Destination node**: The destination node sends ACKs for the received packets and the forwarders who hear the ACK ignore sending any acknowledgment.

Based on the above-mentioned forwarding scheme, the end-to-end delay will be estimated in the “Delay calculation as the routing metric” subsection.

### Data-link layer behavior

Data-link layer behavior is another important subject that has a high impact on channel access delay, and it is fully discussed in this subsection. Since all transmissions in OR schemes are in broadcast mode, data-link layer behavior is different from the standard behavior of IEEE802.11. The first difference is in the contention window adjustment. In IEEE802.11, the contention window starts from a minimum value (CWmin) and doubles on each unsuccessful transmission attempt, while in the broadcast mode, the contention window does not change and remains constant. Therefore, the backoff stage will not be extended. The second difference is in the retransmission behavior. In IEEE802.11, when a sender sends a packet, it will wait for the link-layer acknowledgment from the next-hop node, whereas in the broadcast mode, there is no specific next-hop node and the sender will not wait for the data link layer acknowledgment.

### Delay calculation as the routing metric

Based on the explained forwarding scheme, network layer behavior, and the data link layer behavior, we will calculate the delay that the network imposes on each packet to be transferred between every two nodes as a possible source-destination pair. The calculated delay will form the routing metric and will be used in the CS prioritization. In other words, the delay between the nodes will be assumed as the cost of the routing algorithm. Therefore, each node will form a 2D matrix whose elements are the delays between all possible source-destination pairs in the network. The notations used in the next subsections are introduced in Table 2.

**Table 2:** Notations used in this paper.

Parameter	Meaning
PCS	Potential Candidate Set
*T*_*local*_(*i*, *PCS*)	The delay of delivering a packet from Node *i* to the PCS
*AN*[*i*]	Active nodes from Node *i* point of view
|*AN*[*i*]|	The size of *AN*[*i*] set
*T*_*access*_(*AN*[*i*])	Network access time for Node i
*T*_*trans*_(*data*)	Transmission delay of a data packet
*T*_*ack*_(*i*, *PCS*)	The time required to receive an ACK at Node *i* from the *PCS*
*P*_*success*_(*i*, *PCS*)	The reception probability of Node *i*’s packet by at least one member of its *PCS*
*pdr*[*i*][*j*]	Packet delivery ratio between the Nodes *i* and *j*
*P*_*fwd*_(*i*, *k*, *PCS*)	The forwarding probability of Node *i* packet by Node *k* of the PCS
*t*_*slot*_	The time duration of a slot
*CW*	The selected contention window value
*CWmax*	The maximum value of the contention window
*CWmin*	The minimum value of the contention window
*DIFS*	Distributed Inter-frame space
*Y*_*s*_ (*AN*[*i*])	A random variable describing the interrupts during the *s*-th slot of Node *i*’s backoff slots
*τ*	Attempt probability
*p*	Channel emptiness probability during a given time slot
*q*	Successful transmission probability during a given time slot
*QDU*	Queuing delay unit
*Q*_*i*_	Queue length of Node *i* in QDU
*T*_*CS*_(*i*, *PCS*, *dst*)	The delay of Node *i*’s packet from the *PCS* to Node *dst*
*T*_*e*2*e*_ (*i*, *dst*)	The end-to-end delay between the Nodes *i* and *dst*
*SCS*	Selected candidate set
*Delay*(*i*, *dst*, *SCS*)	The delay of delivering the packet from Node *i* to the *dst*, by *SCS* as its forwarder
*Required_Delay*	Maximum tolerable delay of a packet
*PDR_Tresh*	Packet delivery ratio threshold of the *SCS*’s members
*Delay*_*stc*_	The delay from the source to the SCS
*Delay*_*ctd*_	The delay from the *SCS* to the destination

Suppose that Node *i* wants to send a packet to another node in the network. According to the explained forwarding scheme, Node *i* selects a forwarder set and puts this set in the packet header, and finally broadcasts the packet. it is worth noting that the forwarder set is re-selected for each packet the node wants to forward. Since the results from the delay’s calculation is required for the CS selection, the actual CS is not selected in this step. An abstraction of CS is used for delay estimation. The abstracted CS, called Potential Candidate Set (PCS), contains Node *i* neighbors whose routing metrics for the intended destination are less than that of Node *i*.

Delivering a data packet to the PCS takes as long as the first part of the end-to-end delay, denoted as *T*_*local*_(*i*, *PCS*). In other words, *T*_*local*_(*i*, *PCS*) represents the average delay between the time a packet was sent by Node i and the time of its reception by at least one member of the PCS. *T*_*local*_(*i*, *PCS*) consists of three main parts: channel access time, packet transmission time, and the time needed for the acknowledgment reception.

- **Channel Access Time**: The channel access time depends on the number of network nodes. The more the number of network nodes, the higher the contention level, and subsequently, the higher the access time will be. However, when there are n nodes in the network, the channel access time of Node *i* will not be affected by all the n nodes. Only a subset of the network nodes that are in the transmission range of Node i and have packets to send affects the access time. We define an Active Node set (AN) as a set that includes all these affecting nodes and use *AN*[*i*] to demonstrate this set for Node **i**.
- **Packet Transmission Time**: After accessing the channel, Node i can send the packet.
- **Acknowledgment Time**: after the packet transmission, the sender will wait to receive an ACK from a member of the CS.

Therefore, according to the probabilistic analysis of the number of attempts for a successful transmission [30], *T*_*local*_(*i*, *PCS*) can be calculated as

As the packet’s transmission may be successful after multiple trials, *T*_*local*_(*i*, *PCS*) is multiplied by the inverse of success probability to obtain its average value. For a better explanation, the number of trials to reach success follows the geometric distribution. Therefore, the average number of trials is equal to the inverse of success probability. The calculation of *T*_*access*_(*AN*[*i*]) will be explained later. *T*_*trans*_(*data*) is calculated as

$$\begin{array}{c} T_{local}\left(i,PCS\right)=\frac{T_{access}\left(AN\left[i\right]\right)+T_{trans}\left(data\right)+T_{ack}\left(i,PCS\right)}{P_{success}\left(i,PCS\right)} \end{array}$$ (1)

In Eq 3, *T*_*access*_(*AN*[*i*]) denotes the channel access time for Node *i*, whose active node-set is *AN*[*i*], *T*_*trans*_(*data*) denotes the transmission delay of the data packet, *T*_*ack*_(*i*, *PCS*) represents the time elapsed for Node i to receive an acknowledgment from one of the PCS members, and *P*_*success*_(*i*, *PCS*) is the successful transmission probability, defined as the probability that the packet sent by Node *i* is received by at least one of the PCS members.

As the packet’s transmission may be successful after multiple trials, *T*_*local*_(*i*, *PCS*) is multiplied by the inverse of success probability to obtain its average value. For a better explanation, the number of trials to reach success follows the geometric distribution. Therefore, the average number of trials is equal to the inverse of success probability.

The calculation of *T*_*access*_(*AN*[*i*]) will be explained later. *T*_*trans*_(*data*) is calculated as

$$\begin{array}{c} T_{trans}(data)=\frac{L_{data}}{R} \end{array}$$ (2)

where *L*_*data*_ and *R* represent the packet length and data rate of the link, respectively. *P*_*success*_ (*i*, *PCS*) is calculated using

$$\begin{array}{c} P_{success}(i,PCS)=1-\prod_{j\in PCS}^{}\left(1-pdr\left[i\right]\left[j\right]\right) \end{array}$$ (3)

where *pdr*[*i*][*j*] denotes the PDR between Nodes *i* and *j*. The time duration that Node *i* waits to receive an acknowledgment from one of the members of the PCS, denoted as *T*_*ack*_(*i*, *PCS*), is calculated as

$$\begin{array}{c} T_{ack}(i,PCS)=\sum_{k\in PCS}P_{fwd}(i,k,PCS)*(T_{access}(AN[k])+T_{trans}(Ack)) \end{array}$$ (4)

where *T*_*trans*_(*Ack*) represents the transmission delay of an ACK packet and is calculated by

$$\begin{array}{c} T_{trans}(Ack)=\frac{L_{Ack}}{R}\; \end{array}$$ (5)

where *L*_*Ack*_ represents the length of the Ack message, and *R* shows the data rate. In Eq 4, *P*_*fwd*_(*i*, *k*, *PCS*) is the forwarding probability, of the packet sent by Node *i* being forwarded by Node *k* (a member of the PCS). *P*_*fwd*_(*i*, *k*, *PCS*) can be computed as

$$\begin{array}{c} P_{fwd}(i,k,PCS)=pdr[i][k]*\prod_{j\;\in \;PCS,j<k}\left(1-pdr[i][j\right]), \end{array}$$ (6)

where *pdr*[*i*][*k*] is defined as the PDR in the link between the Nodes *i* and *k* assuming that the PCS members are ordered according to their forwarding priority, and therefore, the lower index shows the higher priority.

*T*_*access*_(*AN*[*i*]), *T*_*access*_(*AN*[*k*]) in Eqs 1 and 4 show the channel access times for Nodes *i* and *k*, respectively. They will be calculated based on the broadcast mode of the IEEE 802.11 DCF standard. In this standard, the time is divided into time slots. We assume that the duration of each slot is *t*_*slot*_ seconds. Generally, when a node has a packet to send, it selects a random number, called contention window (*CW*), from the [0, *CWmax*] interval and waits for an interval called backoff time. The duration of the backoff time is *CW* × *t*_*slot*_ seconds. After waiting for the backoff interval, the node will sense the channel and if the channel remains idle for Distributed Inter-Frame Space (DIFS) time, it will be able to send the packet. If the channel becomes busy during the backoff interval, the node freezes its backoff timer and when the channel becomes idle and stays idle for an extra DIFS time, the backoff timer will resume. It is worth noting that each node is assumed to send only one packet in each channel access round.

According to the above-mentioned process and the corresponding analysis found in [38], the channel access time of Node *i* can be calculated as

$$\begin{array}{c} T_{access}\left(AN\left[i\right]\right)=DIFS+\sum_{s=1}^{CW}\left[t_{slot}+Y_{s}\left(AN\left[i\right]\right)\right] \end{array}$$ (7)

where *Y*_*s*_ (*AN*[*i*]) is a random variable that shows the interrupts experienced by Node *i* within the *s*-th slot of the backoff interval (caused by freezing the backoff timer due to the channel occupancy in this slot), and *AN*[*i*] shows the active node set of Node *i*. *t*_*slot*_ is fixed and does not change in different slots. Also, the expected value of *Y*_*s*_ (*AN*[*i*]) is also independent of s. So, Eq 7 can be rewritten as

$$\begin{array}{c} T_{access}\left(AN\left[i\right]\right)=DIFS+CW*\left(t_{slot}+E\left[Y_{s}\left(AN\left[i\right]\right)\right]\right) \end{array}$$ (8)

where *E*[*Y*_*s*_ (*AN*[*i*])] is the expected value of *Y*_*s*_ (*AN*[*i*]).

As can be seen in Eqs 7 and 8, the channel access time is a function of active nodes. So, it is very important to have a good estimation of the number of *AN*s. Each node should sense the channel, and count the packets sent by the other nodes. If the number of packets of a specific node is greater than a certain threshold, the counter node will consider the corresponding node as an active one. After each calculation period, the node should reset its counters to update the active nodes’ information as much as possible.

To compute Eq 8, *E*[*Y*_*s*_ (*AN*[*i*])]) must be determined. To achieve this, we first determine the Probability Mass Function (PMF) of *Y*_*s*_(*AN*[*i*]). So, a probabilistic analysis is provided for *Y*_*s*_(*AN*[*i*]). Three different states should be considered for the calculation of of the PMF of *Y*_*s*_(*AN*[*i*]):

1. *s*-th slot has no interrupt
2. A successful transmission occurs in the *s*-th slot: the maximum amount of interrupt is equal to *T*_*send*_ = *T*_*trans*_(*data*) + *DIFS*
3. A collision occurs in the *s*-th slot: the maximum amount of interrupt is equal to *T*_*send*_ = *T*_*trans*_(*data*) + *DIFS*

To compute the expected value of the interrupts *Y*_*s*_*AN*[*i*]), we need to determine the probability of each state. In this regard, attempt probability (*τ*) is a pivotal parameter. *τ* shows the packet transmission probability by a node in a given time slot. It is also assumed that the network is in a saturated state and the stations always have packets to send. Since all the transmissions are in the broadcast mode, *CW* values are always uniformly selected from the [0, *CWmax*] interval. Therefore, each node’s attempt probability will be equal to $\frac{1}{CWmax}$. Based on the attempt probability, we provide a probabilistic analysis that is presented in Table 3 [38].

**Table 3:** Probabilistic analysis of Y_s_(*AN*[*i*]).

State	Probability	The interrupt duration
Idle	*p* = (1 − *τ*)^|*AN*[*i*]|^	0
Successful transmission	*q* = |*AN*[*i*]|*τ*(1 − *τ*)^|*AN*[*i*]|−1^	*T*_*send*_
Unsuccessful transmission	1 − *p* − *q*	*T*_*send*_

Table 3 shows the PMF of *Y*_*s*_(*AN*[*i*]). Using the PMF of *Y*_*s*_(*AN*[*i*]), its expected value is calculated as

$$\begin{array}{c} {E[Y}_{s}\left(AN[i]\right)]=0*p+T_{send}*q+T_{send}*\left(1-p-q\right)=T_{send}*\left(1-p\right) \end{array}$$ (9)

which can be simplified as

$$\begin{array}{c} {E[Y}_{s}\left(AN[i]\right)]=Tsend*\left(1-\left(1-{\left(\frac{1}{CWmax}\right)}^{\left|AN[i]\right|}\right)\right) \end{array}$$ (10)

Substituting Eq 8 in Eq 10 one obtains

$$\begin{array}{c} T_{access}\left(AN\left[i\right]\right)=DIFS+cw*[tslot+Tsend*\left(1-\left(1-{\left(\frac{1}{CWmax}\right)}^{\left|AN[i]\right|}\right)\right) \end{array}$$ (11)

Queuing delay also takes a major role in the calculation of the packets’ end-to-end delay. Therefore, it is required to calculate the queuing delay that the packets tolerate at the network nodes. Queuing delay calculation in a multi-traffic flow environment has a high complexity since the nodes have packets from different traffic flows with different QoS requirements. *T*_*local*_(*i*, *PCS*), calculated by Eq 1 will be different for the packets with different destinations and different QoS levels. Therefore, a straightforward mapping between the number of queued packets and the queuing delay cannot be expressed. To overcome this issue, each network node should investigate its queue and calculate the PCS and *T*_*local*_(*i*, *PCS*) for the queued packets periodically. The sum of the *T*_*local*_(*i*, *PCS*) of the queued packets will form the node’s queuing delay. Network nodes should be informed about the other nodes’ queuing delays to calculate the end-to-end delay. However, reporting queuing delays in terms of seconds or milliseconds is challenging due to the precision of the values. So, we define Queuing Delay Unit (*QDU*) as a unit and as a specific constant value (e.g. 0.05ms) so that the nodes express their queuing delays in the *QDU* unit.

Network nodes inform others about their queue length in terms of *QDU*. In this way, network nodes can estimate the queuing delay of each other more correctly.

Now that all the essential parameters of delay estimation have been calculated, the general form of the estimated end-to-end delay can be written as

$$\begin{array}{c} T_{e2e}(i,dst)=Q_{i}*QDU+T_{local}(i,PCS)+T_{CS}(i,PCS,dst) \end{array}$$ (12)

where *Q*_*i*_ is the queue length of Node *i* and the term *T*_*CS*_(*i*, *PCS*, *dst*) shows the delay from the PCS to Node *dst* and can be calculated according to

$$\begin{array}{c} T_{CS}(i,PCS,dst)=\sum_{k\;\in PCS}P_{fwd}(i,k,PCS)*T_{e2e}(k,dst) \end{array}$$ (13)

Based on Eq 12, the delay from Node i to Node dst consists of three parts:

- Queuing delay of Node **i** (*Q*_*i*_ * *QDU*)
- The local delay from Node *i* to the selected PCS (*T*_*local*_ (*i*, *PCS*))
- The expected value of the delay from the PCS to Node *dst* (*T*_*CS*_ (*i*, *PCS*, *dst*))

Therefore, Eq 13 can be used in a Dijkstra-like algorithm to compute the pairwise delay between each pair of nodes. Each network node should have some information to compute the routing metric. This information includes nodes’ queue length and PDR of the network links, which is exchanged in the network by means of a Link-State-like routing protocol. This ensures that the network nodes are aware of all other nodes’ queue lengths and their PDRs, enabling them to compute their routing metric.

### Candidate set selection

The other contribution of this paper is the CS selection method. In multi-traffic flow environments, traffic flows have different required QoS levels. If QoS levels of the traffic flows are not considered in the resource allocation procedure, the network will show similar behavior facing various traffic flows. Therefore, some traffic flows may acquire more network resources than their requirements, while the network is unable to service the other arriving traffic flows. However, if the required QoS levels of the traffic flows are considered, resource allocation efficiency will be improved and more flows will be served in the network. Consequently, considering the traffic flows’ QoS levels in the resource allocation procedure will improve the scalability of the network.

In the context of OR, CS selection is a tool for resource allocation. Therefore, we design our algorithm so that the QoS level of the traffic flow influences its CS selection procedure. In this paper, the CS of a packet changes in each hop. It is assumed that each traffic flow informs its expected QoS level in terms of average throughput. According to the required throughput, the maximum tolerable end-to-end delay that can be imposed on the packets of the traffic flow is computed. This maximum tolerable delay is attached to the packets’ headers. At the forwarding time, each node can obtain the latency applied to the packet by subtracting the timestamp of the packet generation from the current node’s timer, according to which the maximum tolerable delay for the packet is computed. On the other hand, the calculated routing metrics in the previous subsection estimate the delay of the nodes to all possible destinations in the network. Therefore in this step, the CS with the nearest delay to the computed tolerable delay for the packet will be selected as a suitable CS. The problem of optimal CS selection is formulated as a single-objective multi-constraint optimization problem.

$$\begin{array}{c} Min|Delay(i,dst,SCS)-Required\_Delay| \end{array}$$ (14)

s.t:

$$\begin{array}{c} \forall j\in SCS\;\;\;\;\;\;pdr[i][j]\geq PDR\_Tresh>0 \end{array}$$ (15)

$$\begin{array}{c} \forall j\in SCS\;\;\;\;\;\;\;\;T_{e2e}\left(j,dst\right)<\;T_{e2e}\left(i,dst\right) \end{array}$$ (16)

$$\begin{array}{c} \forall j\in SCS\;\;\;\;\;\;Q[j]<Max\_Buffer\_Length \end{array}$$ (17)

In the above optimization problem, the SCS that represents the Selected Candidate Set is the decision variable. In other words, the result of the optimization process will be a value of the SCS that minimizes the objective function. Required_Delay shows the maximum tolerable delay of the packets. The term *Delay*(*i*, *dst*, *SCS*) denotes the delay of delivering the packet from Node *i* to Node *dst* with the use of SCS as the forwarding candidates. *pdr*[*i*][*j*] represents the PDR between the Nodes *i* and *j*, and *T*_*e*2*e*_(*j*, *dst*) is the routing metric of Node *j* for Node *dst* and shows the delay between Node *j* and the possible destination nodes implicitly.

The objective function (Eq 14) minimizes the difference between the maximum tolerable delay and *Delay*(*i*, *dst*, *SCS*). The closer the corresponding delay of the SCS is to the maximum tolerable delay, the better the result will be, and network resources will be assigned to the traffic flows more efficiently. Therefore, resource over-provisioning is avoided.

The first constraint (15) ensures that the SCS members are the neighbors of Node *i*. Furthermore, the PDR between the SCS members and Node *i* should be greater than *PDR*_*Tresh* to reduce duplicate transmissions. The second constraint (16) guarantees that the routing metric between each SCS member and the destination node is less than its corresponding value for Node *i*. Finally, the third constraint(Eq 17) ensures that a node with a full buffer is never selected. Note that *Delay*(*i*, *dst*, *SCS*) is calculated using

$$\begin{array}{c} Delay(i,dst,SCS)={\;Delay}_{stc}+{Delay}_{ctd} \end{array}$$ (18)

where *Delay*_*stc*_ shows the delay between Node i and the selected CS and *Delay*_*ctd*_ represents the average delay from the selected CS to the destination node. In Eq 18, *Delay*_*stc*_ is computed as

$$\begin{array}{c} {Delay}_{stc}=\frac{T_{access}\left(AN\left[i\right]\cup SCS\right)+T_{trans}\left(data\right)+T_{ack}\left(i,SCS\right)}{P_{success}\left(i,SCS\right)} \end{array}$$ (19)

where *T*_*access*_, *T*_*ack*_, and *T*_*trans*_ are calculated based on Eqs 2, 4 and 11, respectively. As expected, Eq 19 is Eq 1 in which PCS has been replaced by *AN*[*i*] ∪ *SCS*.

The union of the active nodes and the SCS can guarantee that a network node will be added to the SCS if its addition decreases the packet transfer time. The addition of the nodes to the SCS, regarding the channel access issues, can degrade the network performance due to the increasing channel occupancy and consequently, the channel access time. However, this issue is avoided in the proposed algorithm, because the overall analysis of the channel occupancy is provided in the form of access delay.

The second term of Eq 18 represents the delay of the CS to the destination and is equal to the expected value of the routing metric to the destination, and can be calculated using

$$\begin{array}{c} Delay_{ctd}=\sum_{k=1}^{\left|SCS\right|}P_{fwd}(i,k,SCS)*T_{e2e}\left(k,dst\right) \end{array}$$ (20)

Note that the optimization problem formulated by Eqs 14–17 is an instance of an optimal subset selection problem. Therefore, any kind of discrete optimization method can be used to find a solution as the best SCS.

**Fig 2:**
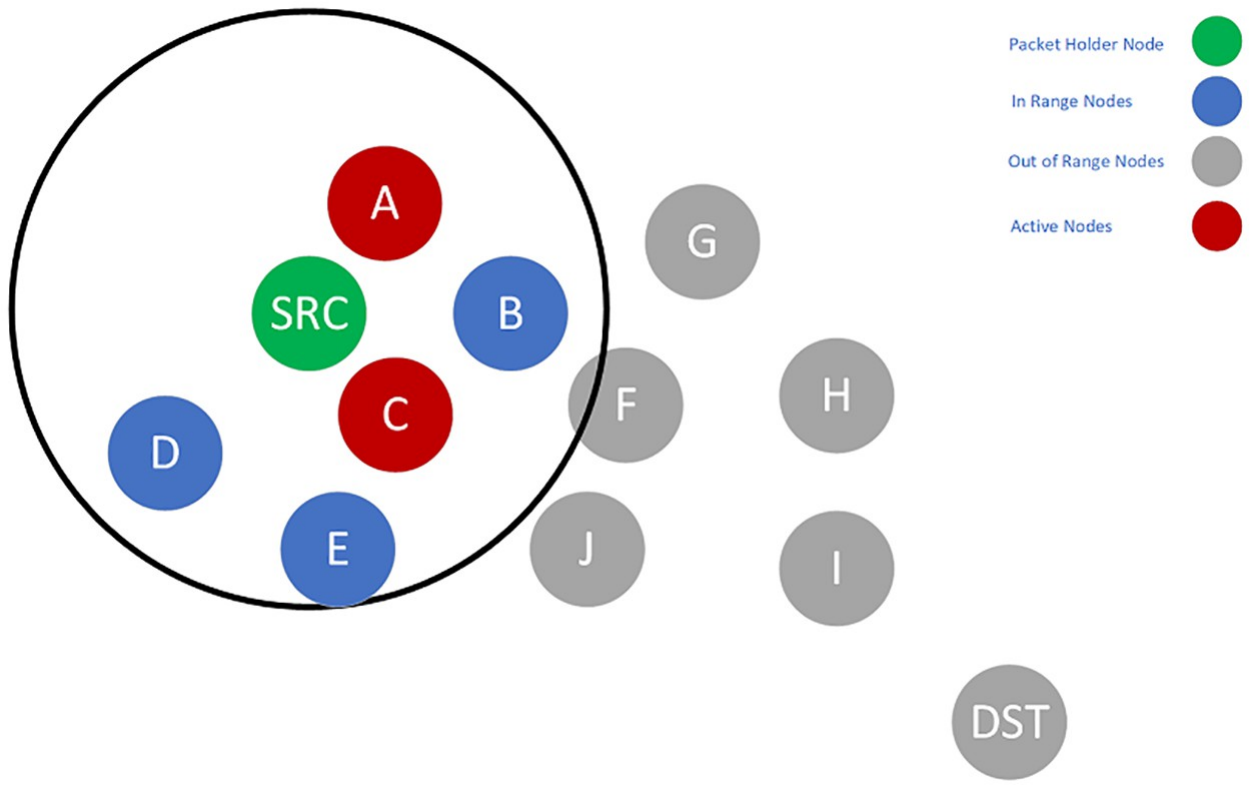
Candidate set selection and packet forwarding of Node SRC.

**Fig 3:**
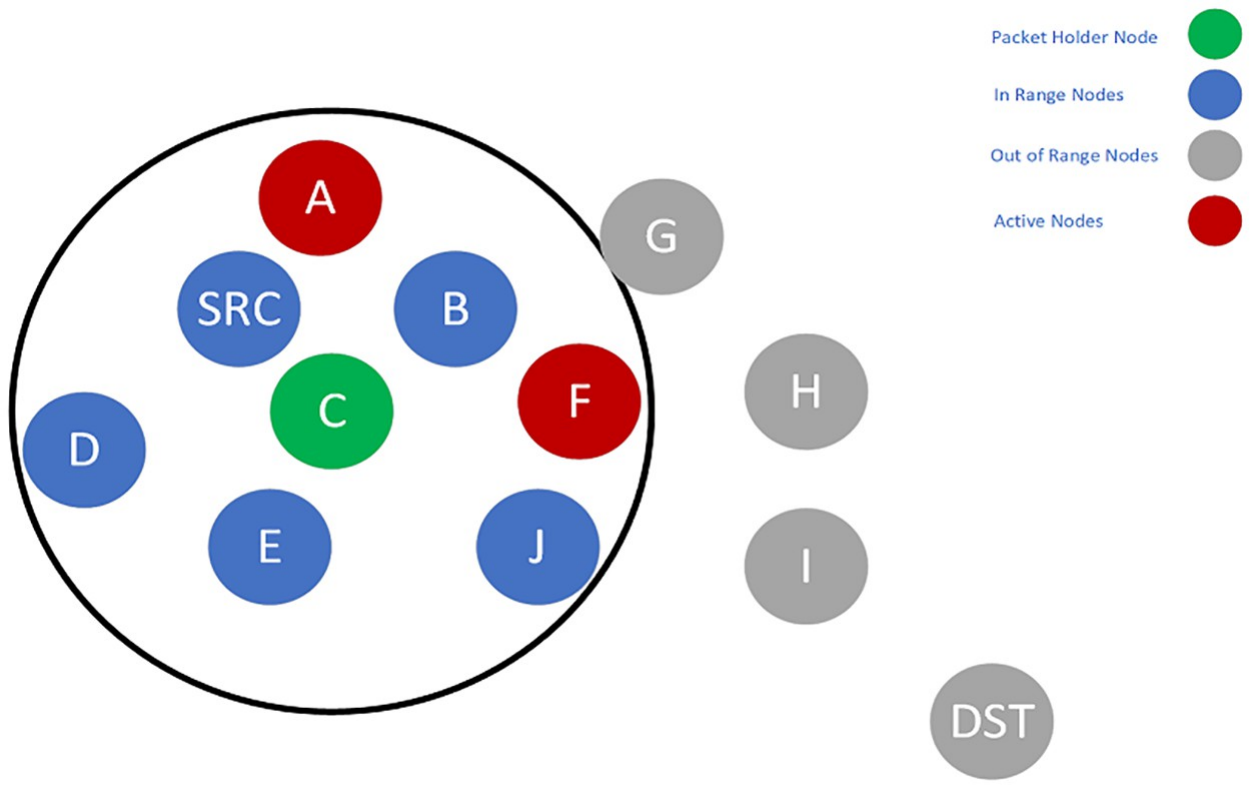
Candidate set selection and packet forwarding of Node C.

**Fig 4:**
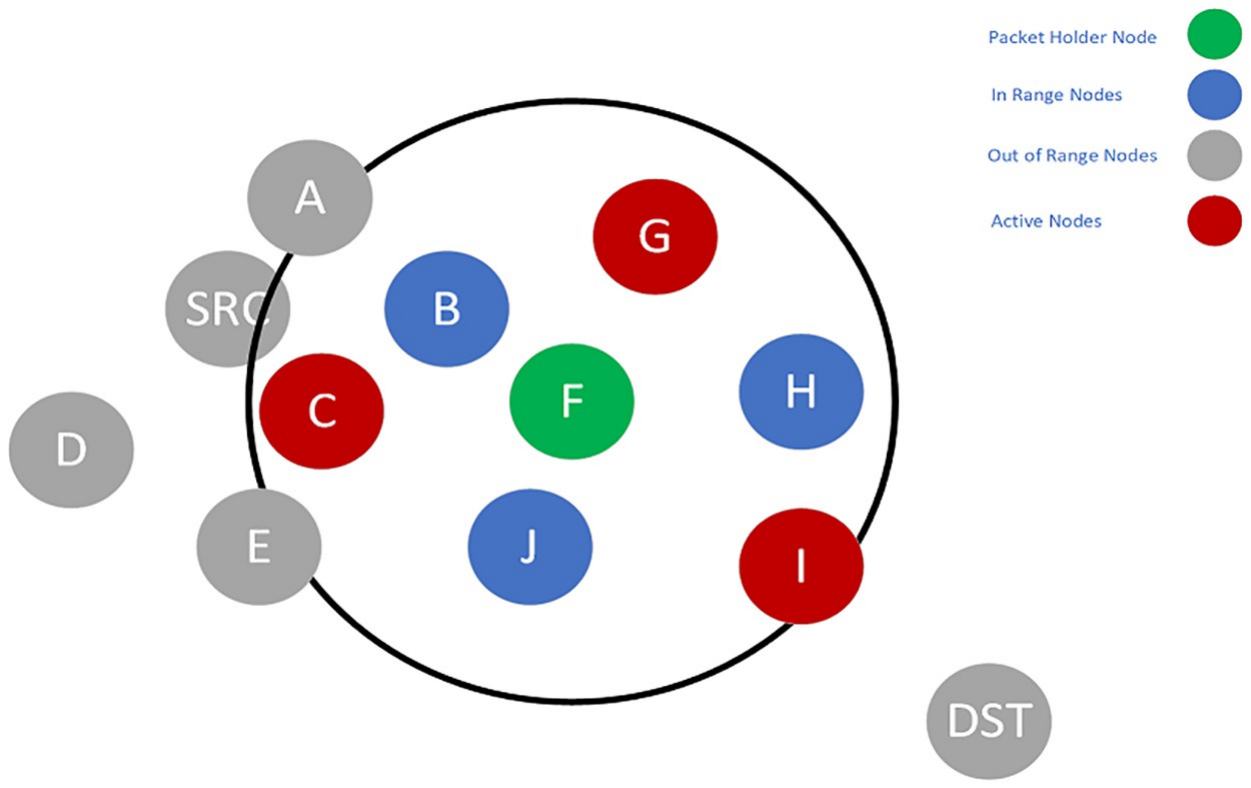
Candidate set selection and packet forwarding of Node F.

**Fig 5:**
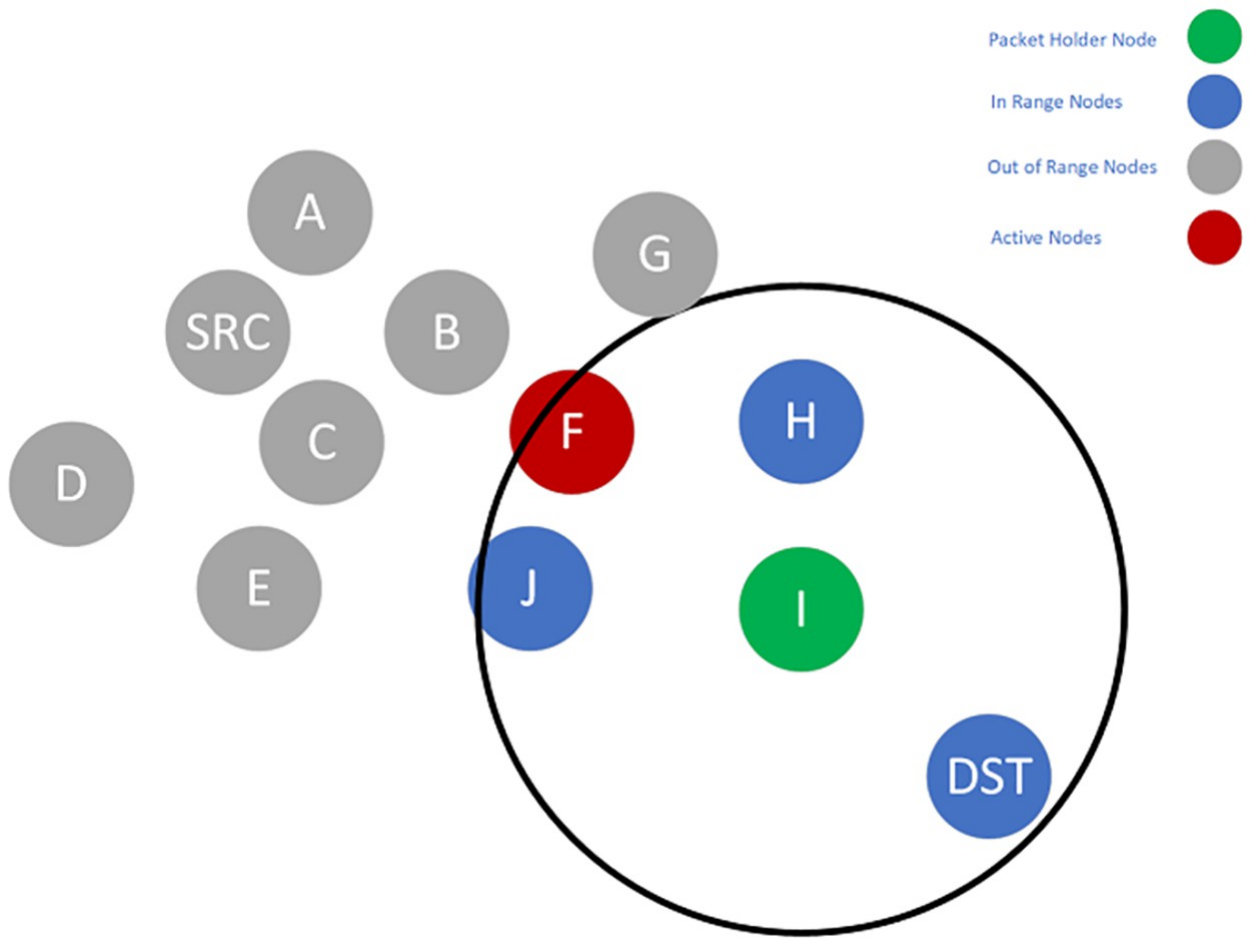
Candidate set selection and packet forwarding of Node I.

To better illustrate how the proposed algorithm works, consider the example shown in Figs 2–5. Suppose Node *SRC* wants to send some packets to the Node *DST*. In Fig 2, which shows the first step of the algorithm, Nodes *A* and *C* are the previously active nodes, and Nodes *F, G, H, I, J*, and *DST* are the *SRC*’s out-of-range nodes.

SRC selects a candidate set, which is a subset of *SRC*’s neighbors. An initial set containing *A, B, C, D*, and *E* is assumed (Fig 2). All of the non-null subsets of this set can be selected as the candidate set. However, each subset is evaluated with the delay imposed on the packets. The subset with the closest delay to the required delay is selected as the final candidate set. For example, one probable choice can be *B*, *D*, *E*. This subset contains no active nodes. Thus, based on Eq 16, this is more probable that the union of this subset with the active nodes leads to higher delay. Another option is the subset of *E*. This single node has a low PDR to the *SRC*. So, in Eq 19, *P*_*success*_(*i*, *SCS*) becomes large, and the overall result leads to a higher delay. Generally, it is more probable that the subsets like *A*, *B*, *C* or *A*, *B*, *D* will be selected because they include the active nodes. Assume that the set of *A*, *B*, *C* is selected, and among the members of this candidate set, Node *C* has the highest priority. So, it will become the packet forwarder and should forward the packet. Node *C*, in a process similar to Node *SRC*, should select the next candidate set from its neighbors (Fig 3) and forward the packet. When Node *C* forwards the packet, Node *F* will become the packet holder and repeat the process. Next, Node *F* selects the candidate set from its neighbors and forwards the packet (Fig 3). Finally, Node *I* becomes the packet holder and selects the next candidate set. Because Node *DST* is the neighbor of Node *I*, it has the highest priority in the candidate set. Therefore, the packet reaches Node *DST* when it is forwarded by Node *I* (Fig 5).

### Cross-layer operations

Another contribution of the proposed algorithm is its cross-layer operation that can improve its performance. The first cross-layer operation is called MAC queue awareness. If a node sends its packet on the channel and does not receive any network-layer ACK from the CS, it will retransmit the packet. This process is necessary for error-prone conditions. When the contention level is very high, the network layer will resend the packet to the MAC layer as it has not been able to access the channel to send the packet and receive its corresponding ACK. Therefore, multiple copies of the packet will appear in the MAC layer queue. To avoid such a situation, the node looks at the MAC layer queue, before the packet retransmission from the network layer. If the node finds the packet in the MAC layer queue, it will refrain from its retransmission. This simple method decreases the number of duplications and improves network performance.

Another simple but influential aspect of our algorithm is the dynamic network layer retry threshold. A high retry threshold may cause more duplications and this will waste network resources. On the other hand, with a low retry threshold, the algorithm may not be able to overcome channel errors. As the network condition is dynamic, a fixed retry threshold is not a suitable option. In our algorithm, the retry threshold of each node will be set based on its SCS. For the SCS, the expected value of the number of transmissions needed to deliver the packet successfully will be selected equal to (*P*_*success*_ (*i*, *SCS*))^−1^ and this amount will be an appropriate choice for the retry threshold of Node *i*.

**Fig 6:**
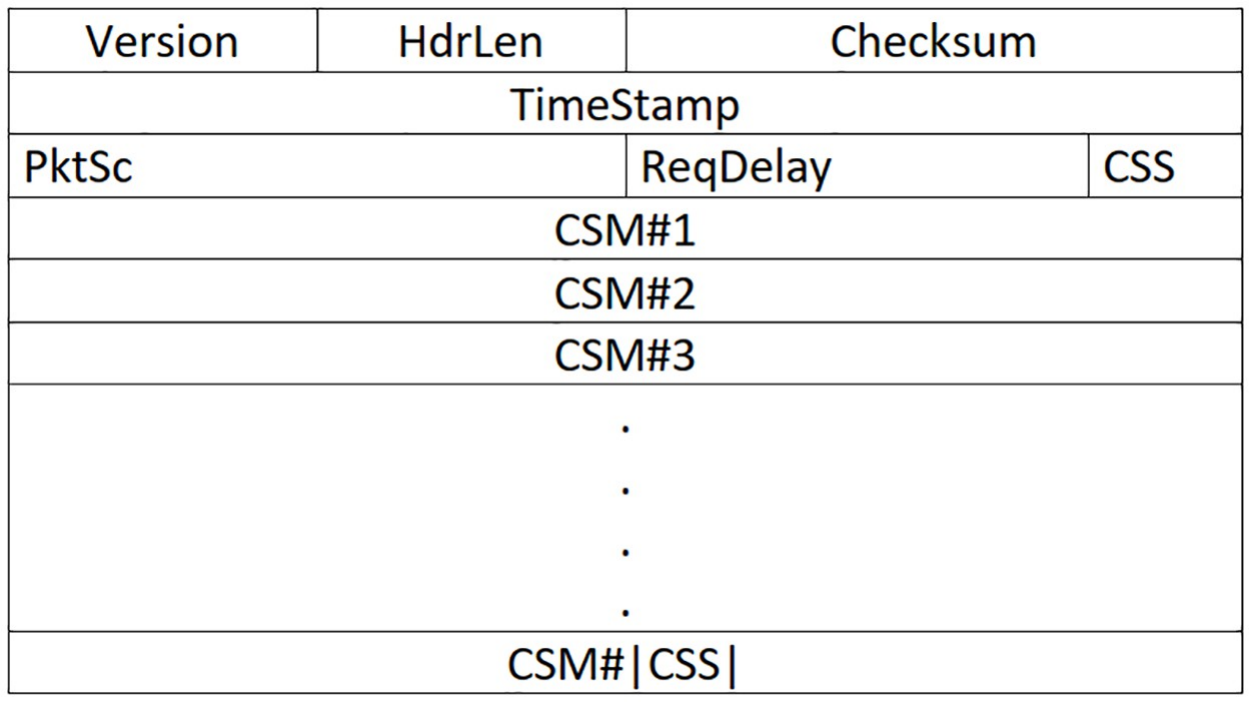
Packets header.

It is worth noting that for the mentioned cross-layer operations, we just need to add some items such as the forwarder set and remaining delay to the header of the packets, and no other changes are needed. To do so, a packet header format is designed to carry the required fields. This header, shown in Fig 6, will be added between the IP header and the 802.11 header. The details of the fields are explained below.

- **Ver**: The version of the proposed algorithm that is necessary to support the backward compatibility.
- **HdrLen**: the header length in terms of the number of 32bit blocks.
- **Checksum**: Checksum of the header fields for error detection.
- **TimeStamp**: The timestamp showing the packet creation time
- **PktSc**: Packet sequence number
- **ReqDelay**: The tolerable delay of the packets. Each forwarder node that receives the packet compares its current time with the TimeStamp field and obtains the delay, experienced so far, and selects the next forwarder set according to the packts’ remaining tolerable delay.
- **CSS (Candidate Set Size)**: The number of candidate nodes. This field will be followed by the IP addresses of the corresponding candidate set members. These nodes are sorted by the decreasing order of the routing metric (highest to lowest priority member).

## Performance evaluation

An extensive simulation study has been done to investigate the performance of the proposed algorithm. For the performance comparison, we selected the works presented in D-ORCD [24] and BORA [26] because of their similarity to our proposed algorithm. Similar to the proposed algorithm, D-ORCD aims at traffic distribution. So, it considers the node’s queue length and the PDR for determining the nodes’ forwarding priority. However, channel contention level status is ignored in the forwarding prioritization. On the other hand, BORA prioritizes the forwarding nodes based on the time in which the network interface is in busy/idle mode. So, BORA is similar to the proposed algorithm in terms of channel contention level status consideration. However, it does not consider the backlogged traffic. In this paper, we consider both backlogged traffic and channel contention level status in the design of the proposed algorithm. Furthermore, D-ORCD and BORA have similar overhead to the proposed algorithm because they also use an ack-based forwarding scheme.

### Simulation setup

The simulation is conducted by the NS-2 network simulator and the simulation parameters are set as shown in Table 4. Each simulation experiment is repeated ten times for validation. The average results of different runs, as well as their 90 percent confidence intervals, are depicted in the figures. Multiple source-destination pairs are selected randomly from the network nodes in each iteration of the experiments. Each source node sends a Constant Bit Rate (CBR) UDP traffic in the network. Furthermore, the experiments are repeated for three different network densities to investigate the effect of the network density on the performance of the algorithms.

**Table 4:** Simulation parameters.

Parameter	VALUE
Simulation area	1000m*1000m
Number of network nodes	10, 15, 20, 50
Nodes’ type	Fixed-mobile nodes
Topology	Random
Transport layer protocol	UDP
Traffic type	CBR
Network’s data transmission rate	Randomly chosen from 2 to 11 Mbps
Physical and data link layer	802.11 ac
Network load	1500 Kbps-7500Kbps
Background Traffic	128 Kbps- CBR
Physical channel model	128 Kbps- CBR
Nakagami-m parameter 1(< 40 m)	*m* = 1.5
Nakagami-m parameter 2(> 40 m)	*m* = 0.75
DIFS	50*s*
Slot time	20*s*
SIFS	10*s*

The Nakagami-m propagation model is considered for the physical channel, to simulate the fading condition. This setting helps to build a lossy network, where the OR algorithm is more appropriate. As mentioned in Table 4, the channel’s fading condition becomes more severe when the communicating nodes get far from each other. More precisely, the fading condition is acceptable for the 0- 40 meter distances, while for distances greater than 40 m, it becomes more severe. The following parameters are used for the comparison and evaluation of the algorithms:

- **Network overall throughput**: defined as the data received by the destinations divided by the elapsed time.
- **Average end-to-end delay**: defined as the average time duration it will take for a packet to be received at the destination.
- **PDR**: defined as the number of packets delivered to the destinations divided by the total number of sent packets.

These three evaluation parameters will be investigated in scenarios with different network offered loads and densities. The offered load shows the total bit rate of the senders, and the network density refers to the number of nodes. Three, four, and five source nodes have been considered for the 10, 15, 20 and 50 node scenarios, respectively. The next three subsections show the results of the simulation in these different scenarios. In the following, we show that the proposed algorithm will improve all performance metrics due to the simultaneous consideration of node queue length as well as the network contention level.

### Throughput analysis

**Fig 7:**
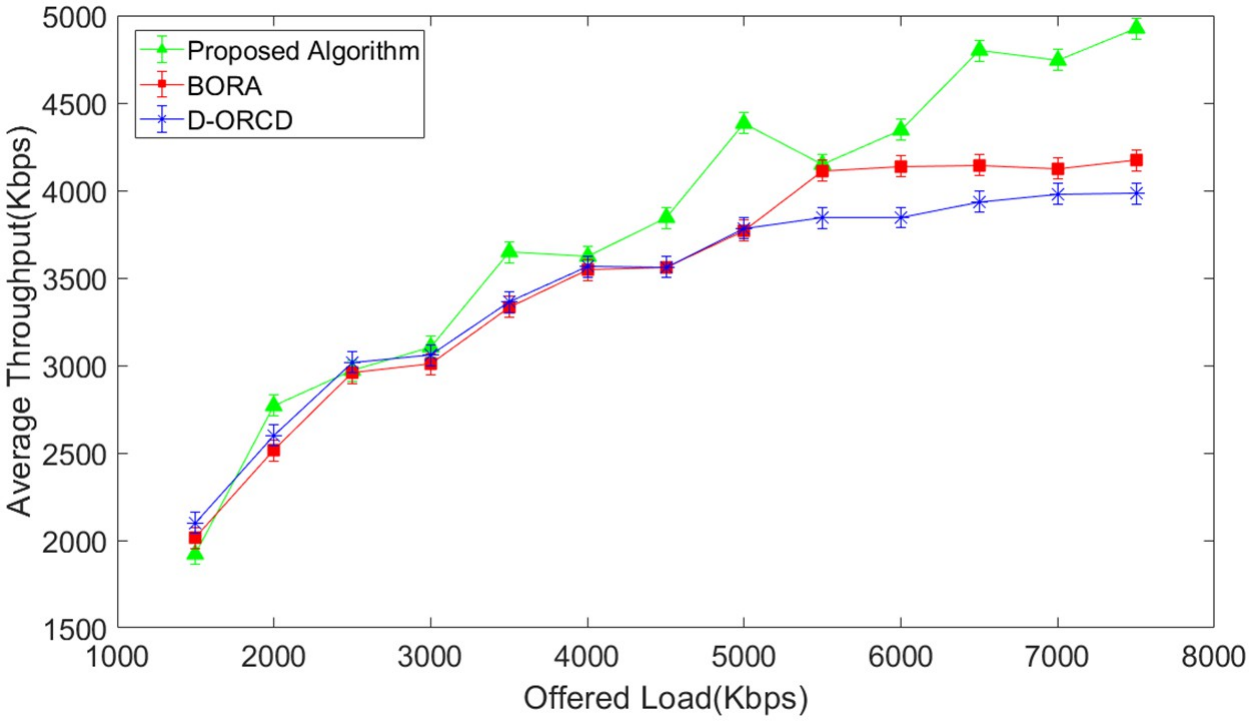
Average network throughput in the 10-node scenario.

**Fig 8:**
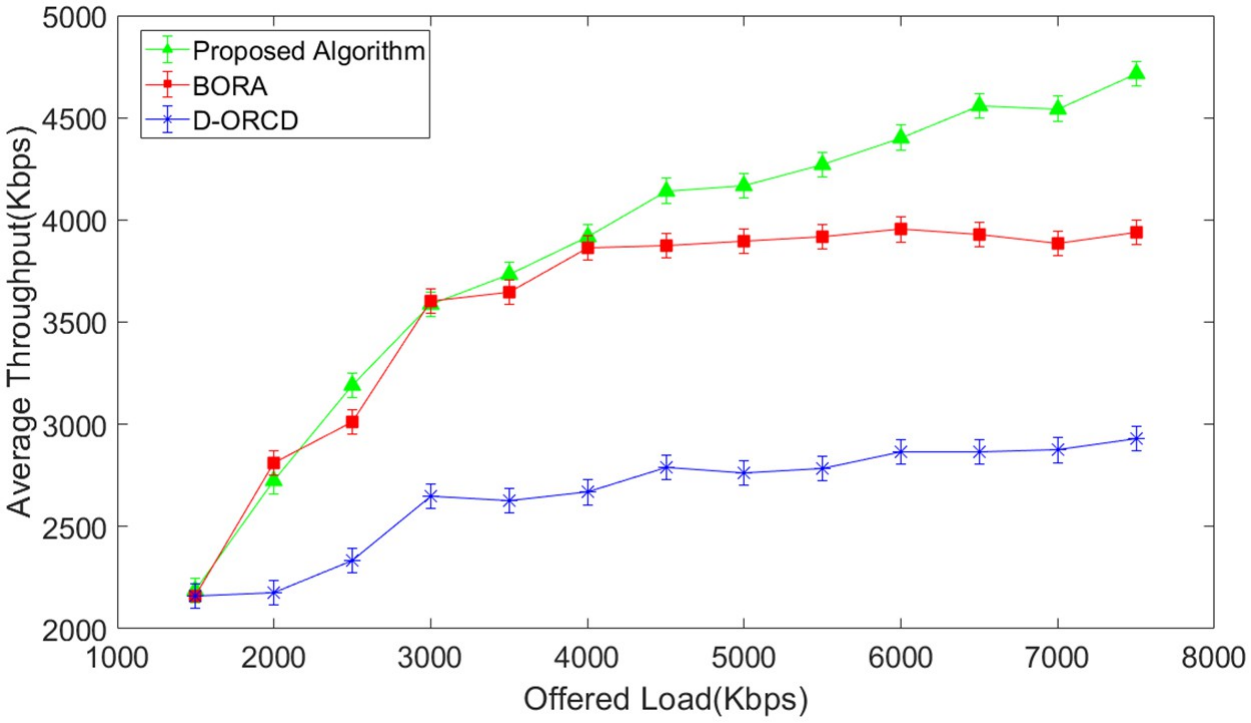
Average network throughput in the 15-node scenario.

**Fig 9:**
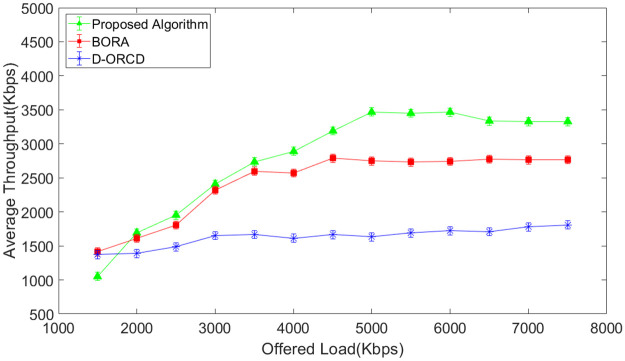
Average network throughput in the 20-node scenario.

**Fig 10:**
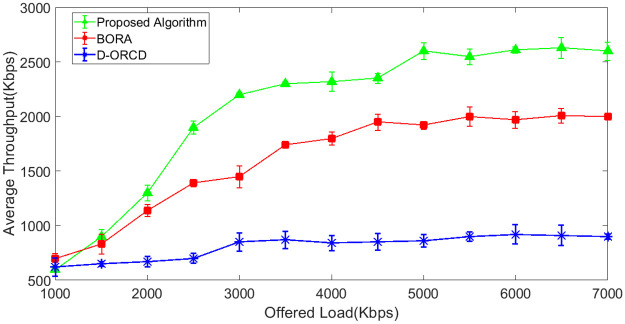
Average network throughput in the 50-node scenario.

The network throughput is evaluated in different network load conditions and for four diverse scenarios with different network node densities. The simulation results are shown in Figs 7–10. It should be mentioned that the analyzed throughput is the goodput of the network, and duplicate packets are eliminated from the throughput calculation. As expected, the throughput increases in UDP-based traffic to a saturation point with the offered load growth. After saturation, the network throughput remains constant. This is well illustrated in Figs 7–10. As shown in these figures, the saturation point of the proposed algorithm occurred at a higher offered network load compared to BORA and D-ORCD. As shown in Figs 7–10, the average throughput of the proposed algorithm is higher than the other two methods for all offered loads higher than 2000Kbps. This improvement becomes more obvious when the network density and also the offered load grow.

This is due to the simultaneous consideration of the channel levels issues like node’s contention, and the nodes’ backlogged traffic. The proposed algorithm performs better than D-ORCD because the nodes’ contention becomes more critical at higher network densities, and our proposed algorithm considers this issue.

BORA that considers channel conditions can partially be adapted to higher contention levels on higher densities and low traffic loads. However, when the traffic load increases, the admission control part of BORA will be activated and prevent the entrance of new traffic flows to the network. So, its throughput remains constant.

**Fig 11:**
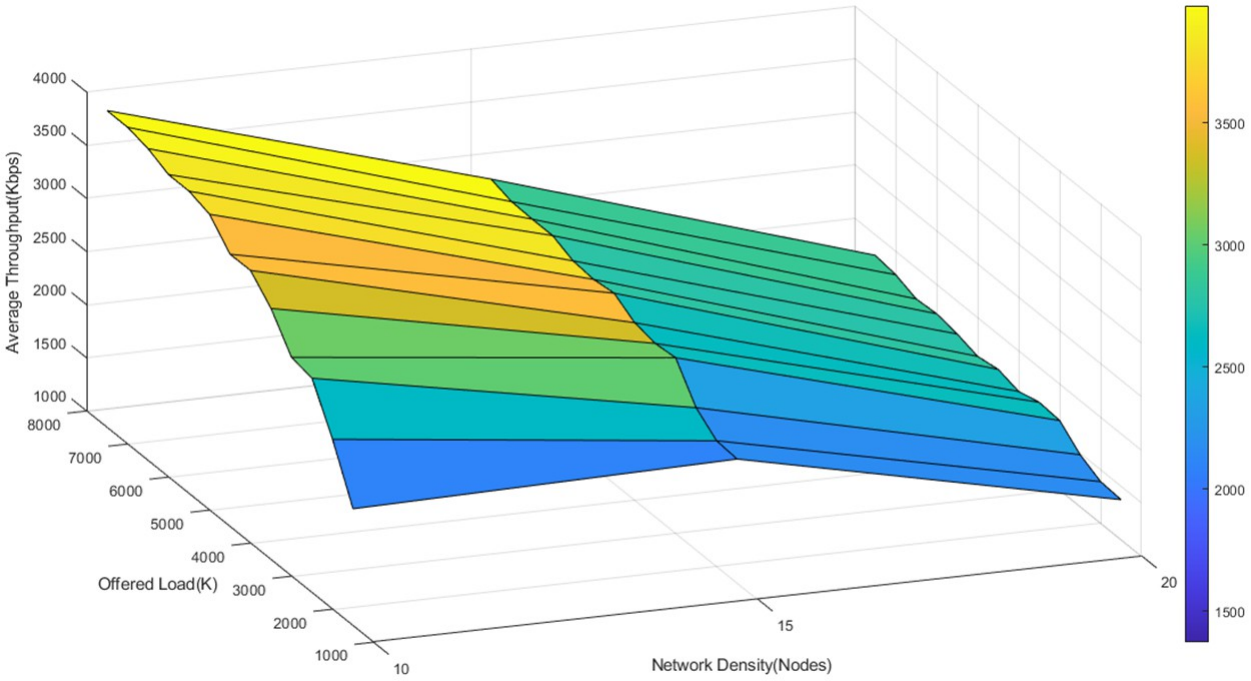
The average throughput of D-ORCD in different network loads and densities.

**Fig 12:**
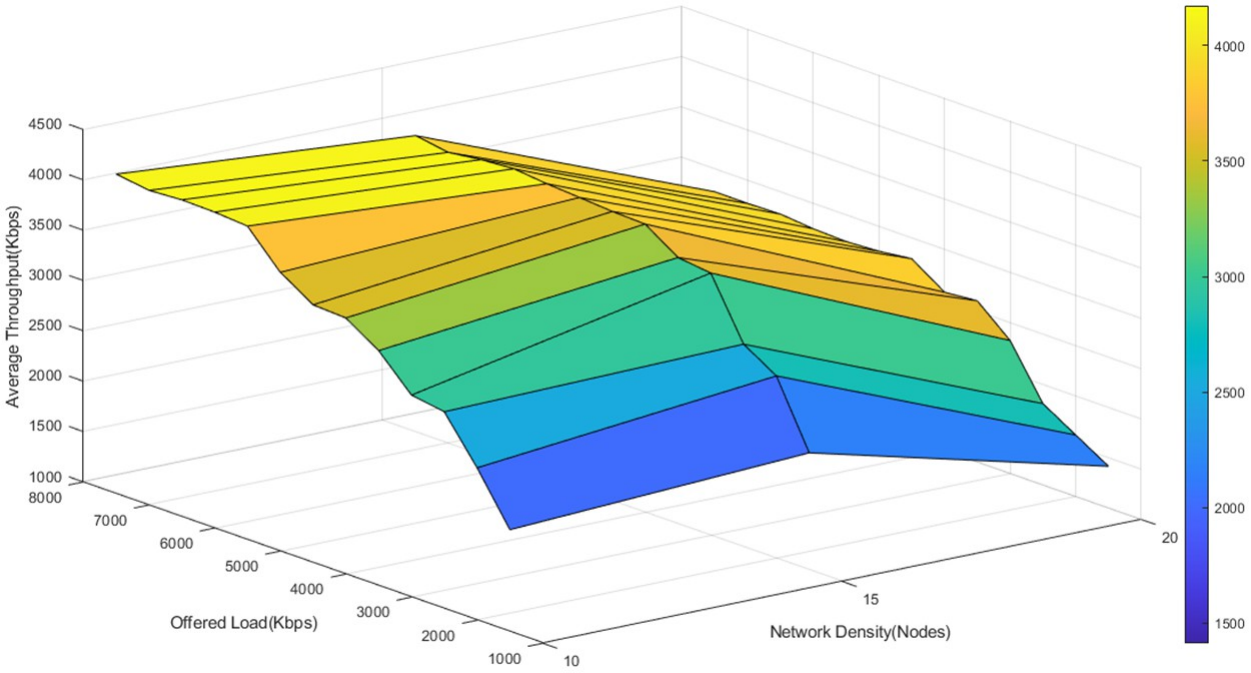
The average throughput of BORA in different network loads and densities.

**Fig 13:**
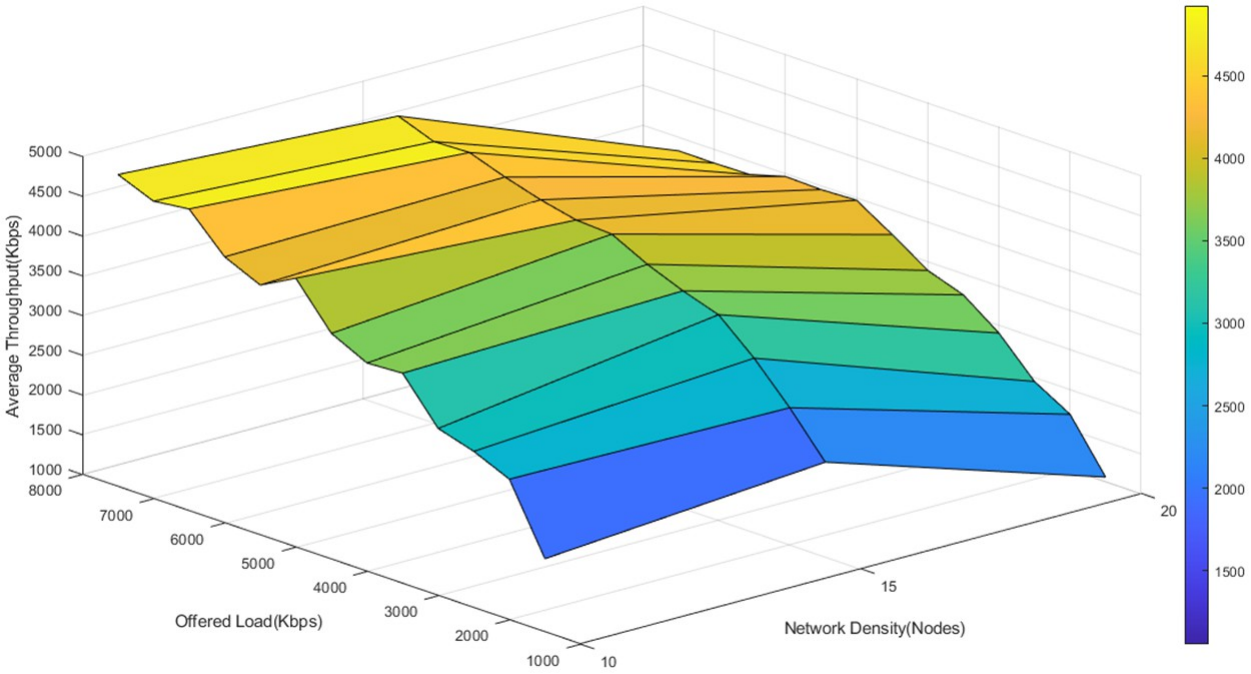
The average throughput of the proposed algorithm in different network loads and densities.

To better understand the fluctuations of the average throughput of the three algorithms in different network loads and densities, the average network throughput is depicted as a function of node density and offered load in Figs 11–13.

As it is shown in Figs 11–13, the average throughput surface of the proposed algorithm is above that of D-ORCD and BORA. It means that the proposed algorithm outperforms the benchmark algorithms in terms of throughput. Also, we expect the performance of the proposed algorithm to be less affected by the increase in the network density. This claim is well supported by the results shown in Figs 11–13. As can be seen in these figures, the maximum throughput of all three algorithms has occurred around 7500 Kbps of the offered load. If 6000 Kbps of the offered load is tracked in various densities, it can be observed in Fig 11 that the throughput of the D-ORCD decreases drastically in high density and this is due to the higher level of inter-node contention that D-ORCD is unable to deal with.

Also, Fig 12 shows that in BORA, the average throughput decreases in high densities and high loads, due to the increasing inter-node contention. In BORA, when inter-node contention becomes more severe, the algorithm reaches the saturation point sooner, and new traffic flows will not be admitted to enter the network. On the other hand, Fig 13 shows that the average throughput of the proposed algorithm decreases slowly from the upper 4500- Kbps area to the 4500-4000 Kbps area. It can be concluded that the proposed algorithm can handle the congestion and inter-node contention well and this will result in better network scalability.

### End-to-end delay analysis

**Fig 14:**
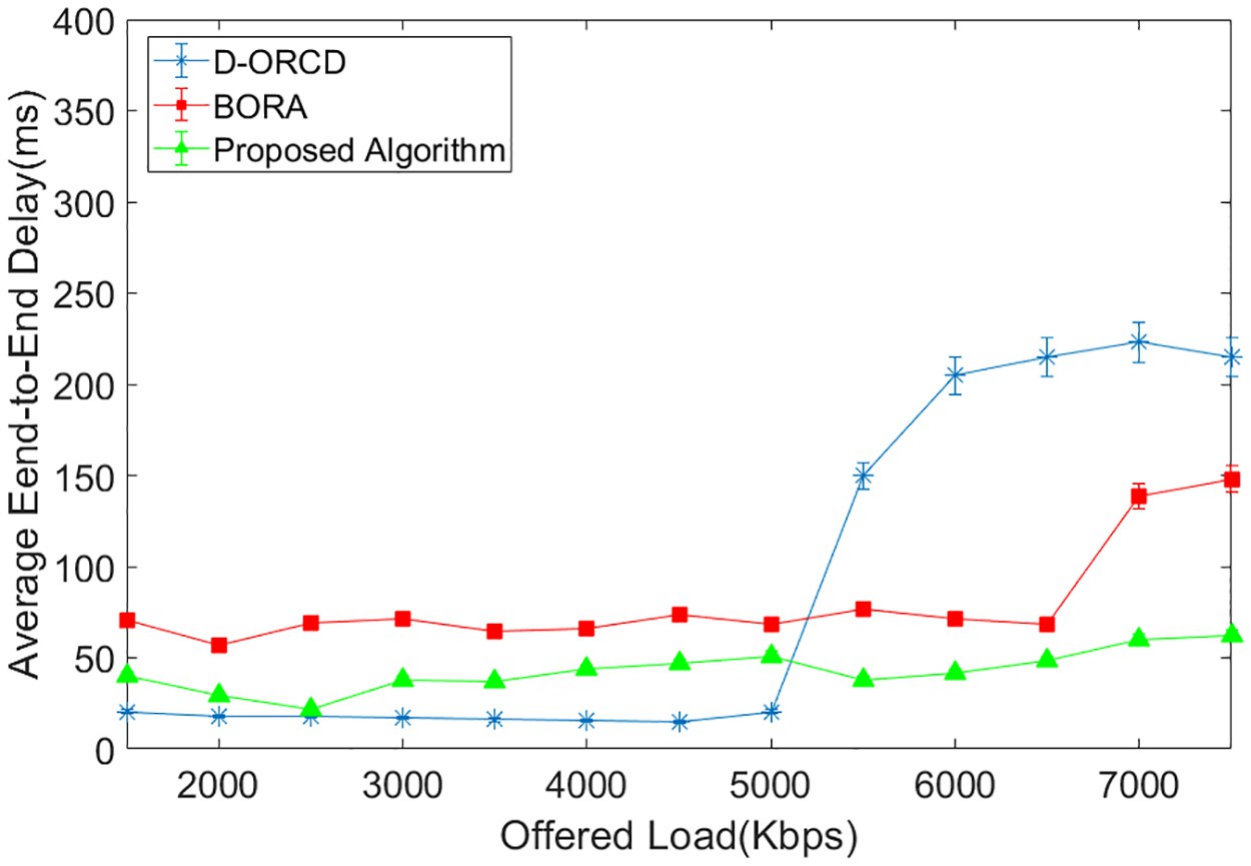
Average end-to-end delay in the 10-node scenario.

**Fig 15:**
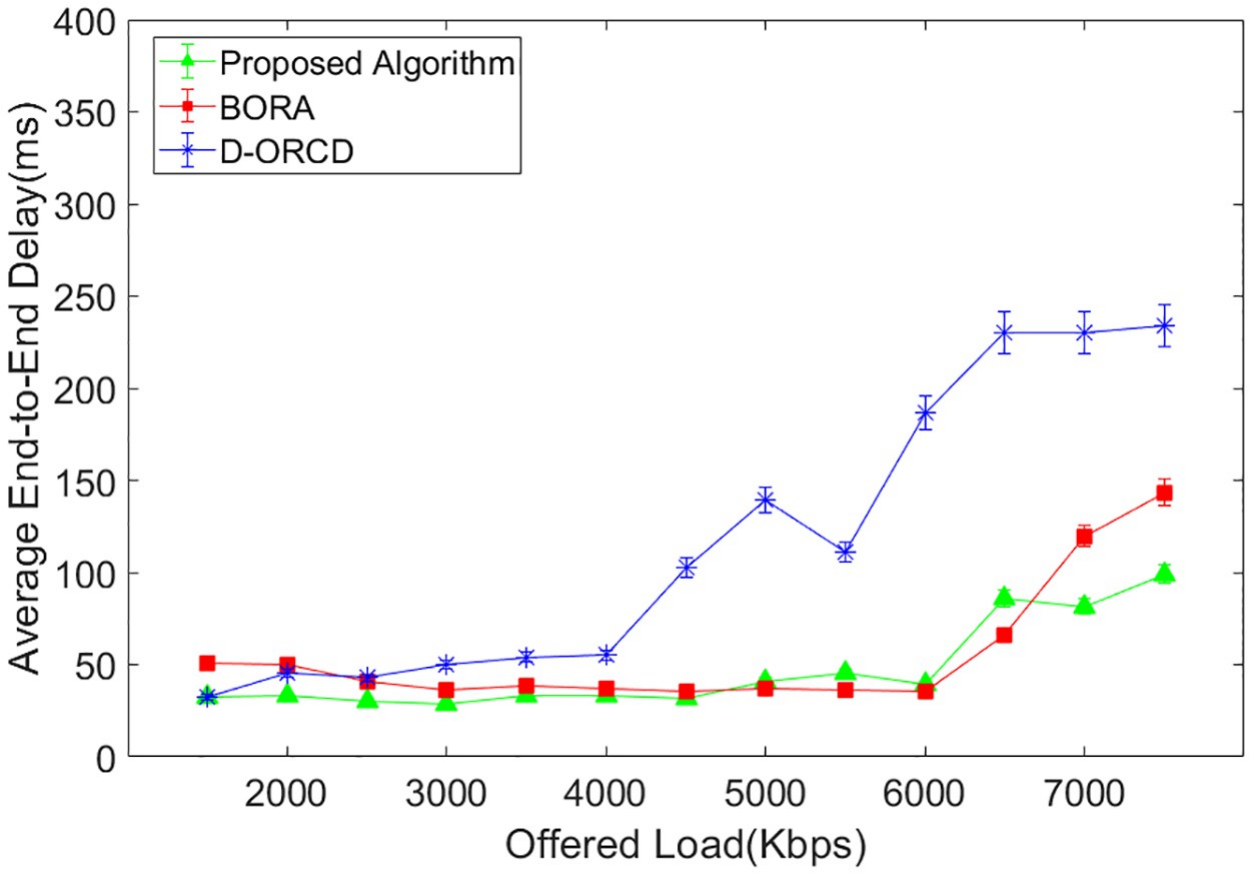
Average end-to-end delay in the 15-node scenario.

**Fig 16:**
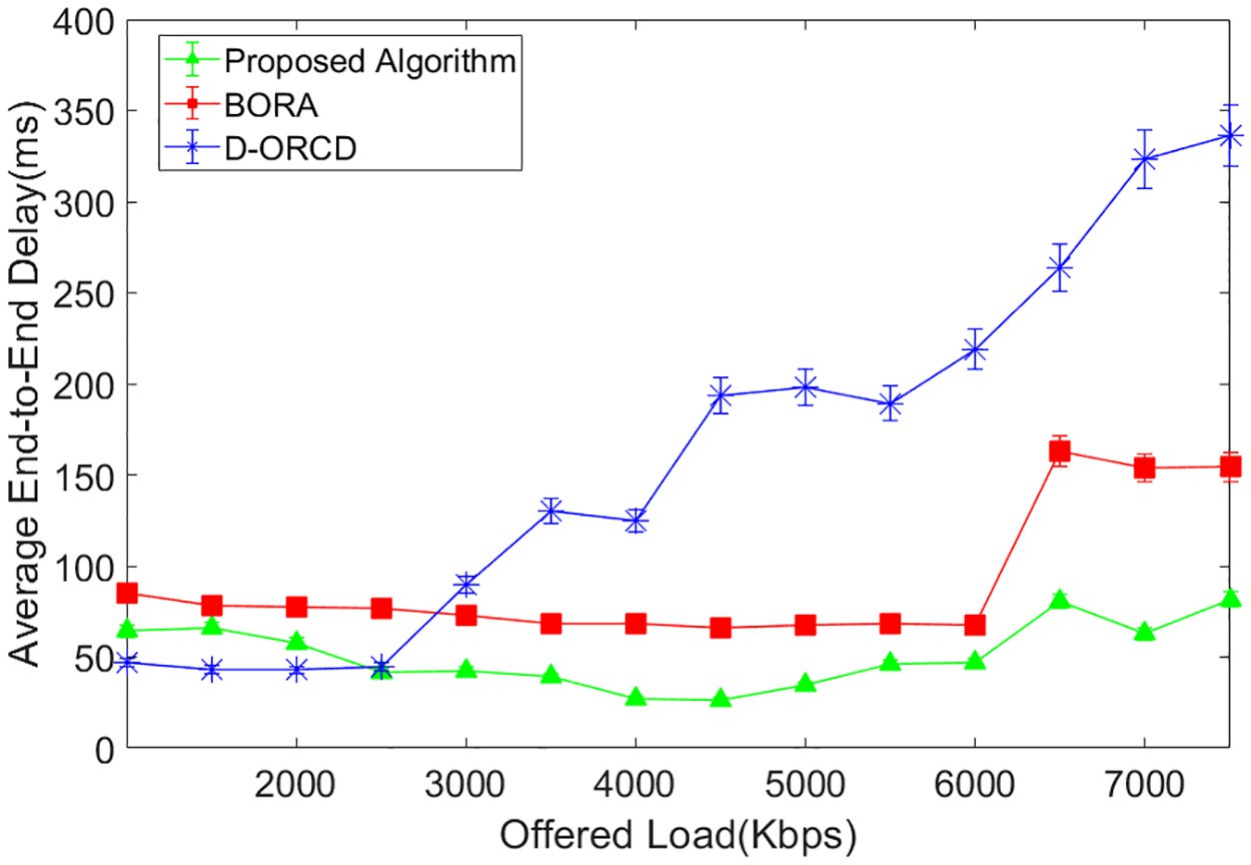
Average end-to-end delay in the 20-node scenario.

**Fig 17:**
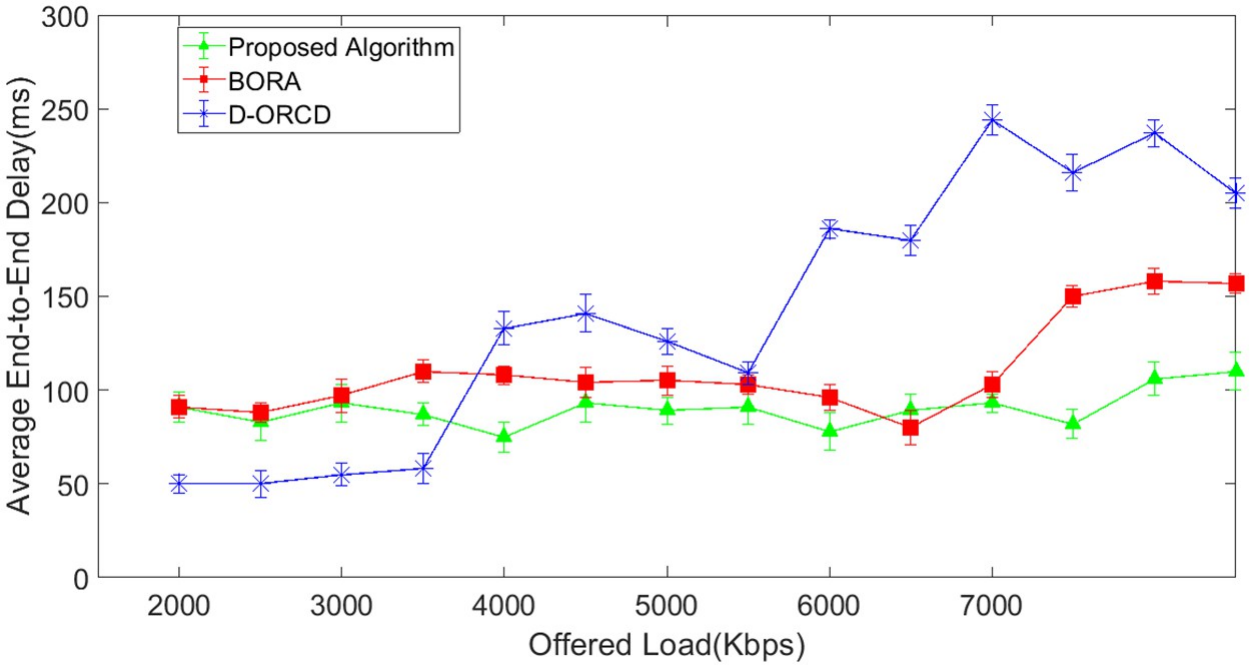
Average end-to-end delay in the 50-node scenario.

Average end-to-end delay is another evaluation parameter that is shown in Figs 14–17. As shown in Figs 14–17, end-to-end delay grows in all three algorithms when the network offered load increases. In D-ORCD, the end-to-end delay has a drastic growth when the offered load is greater than 5000 Kbps. In BORA, this growth occurs around 6500 Kbps of the offered load. However, the increase of the offered load and the density has a low impact on the end-to-end delay in the proposed algorithm. As mentioned earlier in this section, when the network load increases, the network activity level increases as well. In such situations, two types of events occur in the network. The first one is the increase of the inter-node contention and subsequently, the channel access time. Therefore, when a routing algorithm wants to decrease the end-to-end delay, it should first consider the activity level of the network nodes. The second important issue that imposes long delays to the packets is the queue length of the network nodes. BORA can partially handle the contention problem by considering the busy/idle times of the network interfaces, which indicate the network activity level. However, it is unable to handle the congestion because it does not consider the buffer length of the network nodes. So, a packet may go through a node with a long buffer, and subsequently, the end-to-end delay will increase.

On the other hand, D-ORCD is a congestion-aware algorithm. It handles the increase of the queue lengths but is unable to deal with the inter-node contention. So, it shows a good performance in the low levels of network activity. However, the proposed algorithm considers both the queue length and the overall access time in its calculations. Therefore, it performs better than D-ORCD and BORA in terms of end-to-end delay because it can face both congestion and contention at the same time.

### PDR analysis

**Fig 18:**
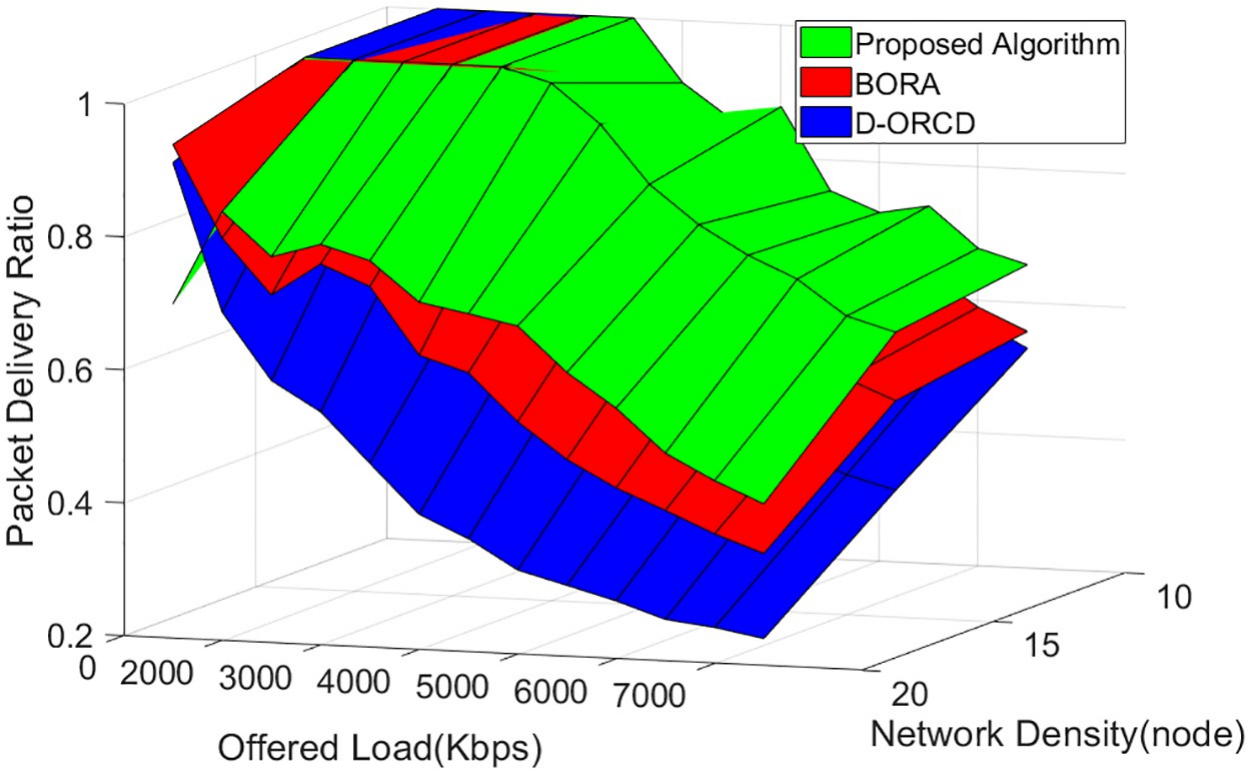
Network scalability in different network densities and different offered loads.

Finally, the PDR of the three algorithms is depicted in Fig 15 at different network densities and different network offered loads. As shown in Fig 18, the PDR of the proposed algorithm is higher for the proposed algorithm compared to BORA and D-ORCD for the majority of all offered loads and densities. We notice that for offered loads greater than 4000 kbps, the proposed algorithm always outperforms BORA and D-ORCD. We also note that the less the PDR of an algorithm is affected by the increase in network density or network offered load, the more scalable it is. Therefore, it can be concluded that the proposed algorithm also outperforms D-ORCD and BORA in terms of scalability. The scalability improvement is because of the overall insight into the channel-level condition that is considered in our algorithm, where the number of active nodes will increase if it is useful to the entire network and not just to a single traffic flow.

### Complexity analysis

This subsection provides a brief analysis of the time complexity of the proposed algorithm as well as D-ORCD and BORA. Routing metric computation and candidate set selection are the two main components of the OR algorithms. Therefore, the time complexity of these components will be investigated. In the routing phase of our algorithm, each node computes the routing metric with a distributed Dijkstra-like algorithm. Therefore, its time complexity will be *O*(*V*^2^), where V represents the number of nodes. BORA and D-ORCD also have the same complexity in the routing phase because they use similar strategies. On the other hand, candidate set selection is an instance of optimal subset selection. In our algorithm, the brute force algorithm is used in this step, in which all subsets of PCS must be explored to find the best answer. Thus, the complexity of the candidate set selection is *O*(2^*N*^). BORA and D-ORCD also need to compute the delay of all potential forwarder sets in their candidate selection phase. Therefore, for both BORA and D-ORCD, the time complexity of this phase is *O*(2^*N*^) as well. Hence, we conclude that the complexity of our proposed algorithm is the same as the complexity of the benchmark algorithms.

## Discussion

Based on the simulation results, it can be concluded that the performance metrics of the network using the proposed algorithm are improved compared to the benchmark algorithms. However, the complexity remains unchanged. Thinking of the reasons for these improvements leads us to the following points: When lots of traffic enters the network, network congestion may occur, where the queue length of network nodes is long. Therefore, the packet delay becomes higher. Congestion control mechanisms try to pass the traffic along less congested paths. However, for a network with random medium access mechanisms, like IEEE 802.11, it will be possible that a node with a short queue imposes long delays to the packets due to the severe inter-node contention for accessing the shared medium. The proposed algorithm aims to consider both issues on one side and the QoS requirements of the traffic flow on the other side. The proposed method provides the flows’ QoS by spending adequate resources, which leads to better utilization of the network resources too. D-ORCD focuses on the queue length, and BORA only considers the medium access contention. Furthermore, none of them pays any attention to the required QoS level of the traffic flows.

## Conclusion

This paper proposes a new cross-layer QoS-aware OR that aims to control network congestion by taking into consideration the backlogged traffic, the traffic flows’ QoS level, and the channel occupancy rate. Conventionally, the backlogged traffic is used as the congestion indicator, and congestion control algorithms conduct the traffic over the paths with lower overall backlogged traffic. Even though backlogged traffic consideration is essential in the congestion control process, some other issues such as channel contention level status can also lead to network performance degradation. Therefore, backlogged traffic, channel occupancy, and the QoS levels of traffic flows have been considered together in this paper in the forwarder nodes’ selection and prioritization procedure with the aim of traffic distribution in the network. The usage of both backlogged traffic and channel occupancy makes traffic distribution more efficient. Also, the traffic flows’ QoS level consideration makes the algorithm assign resources to the traffic flows sufficiently and the potential network capacities will be utilized. Some cross-layer ideas (MAC queue awareness and dynamic retransmission threshold) have also been applied to prevent network resource wastage, resulting in improved network capacity. Simulation results showed that the proposed algorithm improves the network performance in terms of average throughput, end-to-end delay, PDR, and network scalability, especially in high load conditions.
