# Correction: Joint congestion and contention avoidance in a scalable QoS-aware opportunistic routing in wireless ad-hoc networks

**DOI:** 10.1371/journal.pone.0344421

**Published:** 2026-03-05

**Authors:** Ali Parsa, Neda Moghim, Sasan Haghani

The following information is missing from the Funding statement: The work of Sasan Haghani was supported by the National Science Foundation under Grant HRD2011689.

There are a number of content errors in Table 4. Please see the correct Table 4 here.

**Table 4:** Simulation parameters.

Parameter	Value
Simulation area	1000 m*1000 m
Number of network nodes	10, 15, 20, 50
Nodes’ type	Fixed-mobile nodes
Topology	Random
Transport layer protocol	UDP
Traffic type	CBR
Network’s data transmission rate	Randomly chosen from 2 to 11 Mbps
Physical and data link layer	802.11 ac
Network load	1500 Kbps-7500 Kbps
Background Traffic	128 Kbps- CBR
Physical channel model	Nakagami
Nakagami-*m* parameter 1(< 40 m)	*m* =1.5
Nakagami-*m* parameter 2(>40 m)	*m* = 0.75
DIFS	50 µs
Slot time	20 µs
SIFS	10 µs
